# Commentary: Past, present, and future of epigenetics applied to livestock breeding — Hard versus Soft Lamarckian Inheritance Mechanisms

**DOI:** 10.3389/fgene.2016.00029

**Published:** 2016-02-23

**Authors:** Edward J. Steele

**Affiliations:** CY O'Connor ERADE Village FoundationPiara Waters, WA, Australia

**Keywords:** epigenetics, animal breeding, population genomics, sustainability, breeding programs, Lamarckian inheritance

The article by González-Recio et al. (2015) claims to “review the concept of Lamarckian inheritance and the use of the term epigenetics in the field of animal genetics.” I began reading with interest as I am involved in selective livestock improvement (Williamson et al., 2011) using the ancestral haplotype approach to establish associations with desirable beef quality traits (Dawkins, 2015). I was curious where the epigenetics field was situated in this regard. However, in the introductory section, “The Old Ideas,” I considered their comments on an earlier book of mine (Steele et al., 1998) were incorrect. Further, the rest of their article had, in my view, a major omission on what constitutes *Weismann's Doctrine* and the Central Dogma of Molecular Biology (below). Having said this the review by Gonzalez-Recio et al is an otherwise thoughtful and informative article on the application of transgenerational epigenetic ideas and phenomena to livestock improvement. Indeed I have no argument with accurate recounting of the difficulties documenting “hard” epigenetic inheritance in mammals (the transmission of an epigenetic character beyond three generations).

Understanding the genetic rules for how a reversible (erasable and thus “soft”) epigenetic trait can be made into a “hard” genetic transmission process involving modifications to germline DNA sequences, is a worthy research goal. Given the current state of immunological knowledge I am persuaded by the data that “hard” types of soma-to-germline transfer are ongoing at very high frequency in human immune system germlines, and, by extension, other mammalian germlines (below).

I have been developing Lamarckian soma-to-germline concepts and reverse transcriptase (RT) -based feedback mechanisms—“hard” Lamarckian Inheritance—since 1978 (Steele, 1979, 2009a; Rothenfluh and Steele, 1993; Rothenfluh et al., 1995; Blanden et al., 1998; Steele et al., 1998; Zylstra et al., 2003; Steele and Lloyd, 2015). This research has run parallel to investigations on similar RT-based mechanisms in the antigen-driven somatic hypermutation (SHM) of rearranged immunoglobulin (Ig) variable genes, so called VDJs (Steele and Pollard, 1987; Blanden et al., 1998; Weiller et al., 1998; Franklin et al., 2004; Steele et al., 2006; Steele, 2009b). This has led to studies showing that RT-based strand-biased mutation mechanisms, and recently to Robyn Lindley's codon-contexted targeted mutagenesis, apply to *dysregulated* SHM as a general causal mechanism in all cancers (Steele and Lindley, 2010; Lindley, 2013; Lindley and Steele, 2013).

Given that reverse transcription is at the heart of any modern “hard” Lamarckian inheritance mechanism I was surprised that “reverse transcription” is not mentioned by Gonzalez-Recio et al. This is curious because the RNA to DNA step has been embodied in the Central Dogma of Molecular Biology since the discovery of reverse transcription by Temin and Mizutuni (1970) and Baltimore (1970). Crick, who like Temin anticipated it enshrined it in his famous “modification” of the Central Dogma in *Nature* in 1970 (Crick, 1970; summarized in Figure 1A).

**Figure 1:**
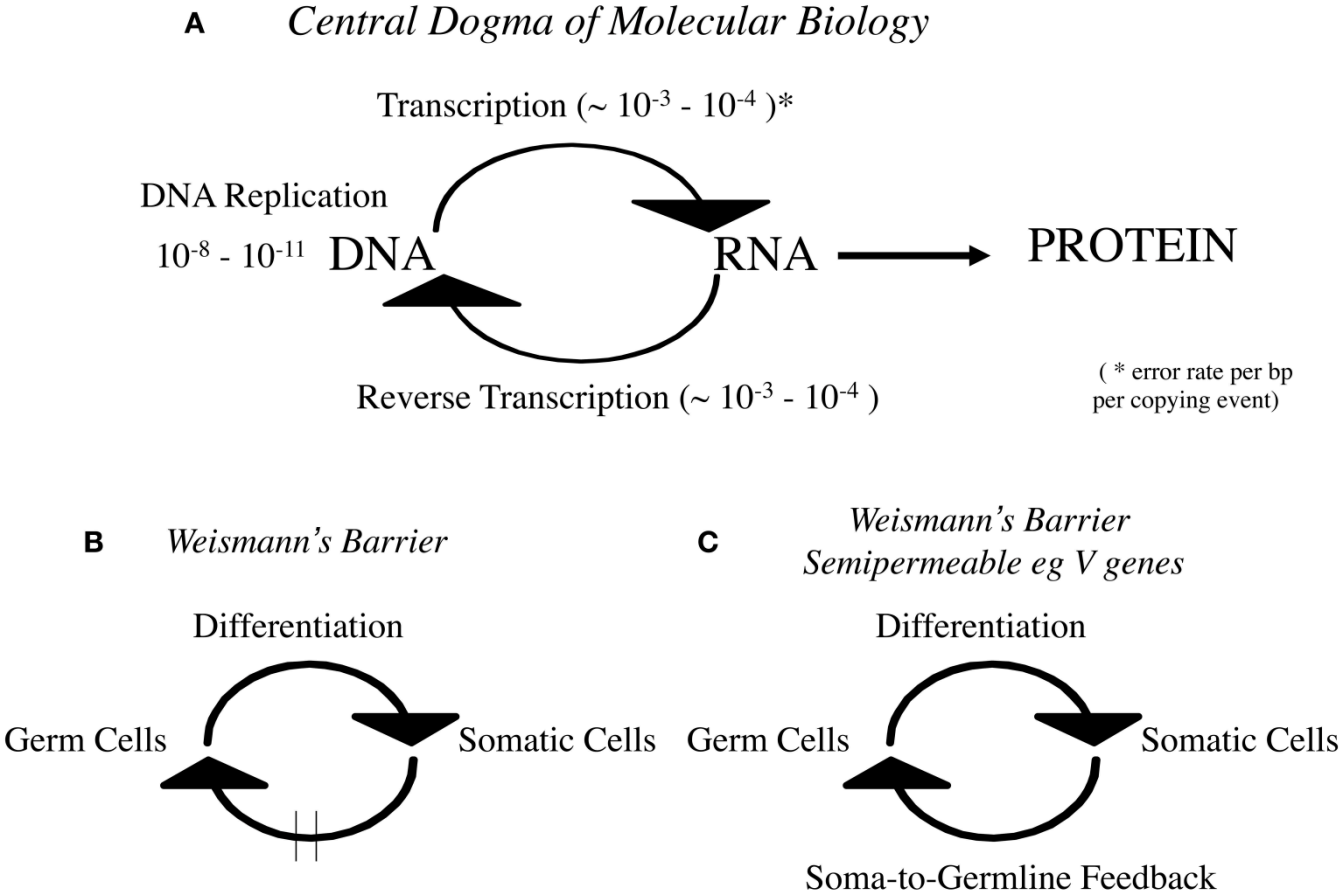
**(A)** Central Dogma of Molecular Biology, **(B)** *Weismann's Barrier*, and **(C)** *Weismann's Barrier is semipermeable eg V genes*.

Indeed the rigid dictum DNA->RNA->Protein is the earlier 1960s rendition which is often mistakenly confused with *Weismann's Doctrine* (Figure 1B). It must be made clear that *Weismann's Barrier* enshrines a *cellular theory* of information flow whereas the Central Dogma is a theory of information flow at the *molecular level*. I found it necessary to draw these clear distinctions when the *Somatic Selection Hypothesis* was first formulated 37 years ago (Steele, 1979). In that theory the data were marshaled to advocate that for the immune system at least, *Weismann's Barrier* was selectively permeable to somatic immunoglobulin V gene mutants (Figure 1C).

I draw out these historical threads as they were not made clear in the Gonzalez-Recio et al article.

My primary reason for writing this invited Commentary is the statement by the authors: “In immunology, Steele et al. (1998) claimed that environment could make the immune system to change its DNA structure, and these changes could be transmitted to the offspring, assertions that have yet to be confirmed.” Apart from anything else I might add our work ca. 1998 was more than just an “assertion,” as it summarized 20 years of work and experimental data (note: “Basic Books” as mentioned in the authors' reference list were not the publishers of *Lamarck's Signature* in the US, it was Perseus Books).

Extensive DNA sequence data shows that the signature of antigen-driven somatic hypermutation of somatically rearranged VDJ genes is embedded within *all* vertebrate *unrearranged* germline V segment arrays examined (Rothenfluh et al., 1995; Blanden et al., 1998; Steele, 2009a; Steele and Lloyd, 2015). This striking fact requires a rational explanation—we have provided that explanation and this has *never* been challenged by molecular immunologists over the past 25 years (at least since our first published report on such patterns in Rothenfluh and Steele, 1993). The logic of this interpretation is outlined in our many papers and the 1998 book, *Lamarck's Signature*.

Certainly as Fogarty (2002) and the group of Corrado Spadafora have repeatedly shown (Zoraqi and Spadafora, 1997; Spadafora, 2008; Cossetti et al., 2014) there is *no physical barrier* preventing somatic RNA/DNA sequences entering the mammalian germline. Sperm developing in the epididymis are most susceptible to this uptake. The transfer of somatic regulatory miRNAs may well use the same soma-to-germline channel for epigenetic transfers in male mice (Rassoulzadegan et al., 2006). This is now emphatically underlined by the recent work of Oliver Rando and colleagues which clearly shows that “small RNA biogenesis and its dietary regulation during post-testicular sperm maturation” linking these “tRNA fragments to regulation of endogenous retroelements active in the preimplantation embryo” (Sharma et al., 2016). Thus vesicles identified as “epididymosomes” carrying RNA payloads matching those of mature sperm, clearly fuse with spermatozoa during epididymal transit and can also be shown to deliver these somatic RNAs to immature sperm *in vitro* (Sharma et al., 2016). What is lacking in all this is a reverse transcription step to lock in these somatic RNAs into germline DNA. Likely RT candidates are the Y family of DNA polymerases (eta, kappa, and iota) or LINE retroelement encoded reverse transcriptases (Franklin et al., 2004; Spadafora, 2008).

However, RNA-templated DNA repair is a generic process in Eukaryotes feeding RNA information back to DNA by conventional DNA polymerases. Thus there exists in the yeast *Saccharomyces cerevisiae* a default DNA repair pathway whereby normal DNA replication polymerases use short RNA templates (6–12 nt) to repair double strand breaks in a physiologically efficient manner (Storici et al., 2007).

Finally there is now a clear necessity to seriously consider non-mendelian soma-to-germline transfer modes. Individual human antibody V repertoire sequencing and V-D-J haplotype analyses are now showing that novel V genes are unexpectedly appearing *at a very high rate* in the human germline implying that such events happen prior to *every* sperm—embryo union [critically evaluated in Steele and Lloyd (2015) but see in particular Kidd et al. (2012) and Gadala-Maria et al. (2015)]. This striking realization throws down the gauntlet to develop more reliable and accurate long-read DNA sequencing so that *complete haplotypes* in the megabase range at the IGHV and IGHL loci—which contain highly similar V sequences—can be characterized by testing for putative haplotype segregation in three generation families (Steele and Lloyd, 2015).

## Author contributions

The author confirms being the sole contributor of this work and approved it for publication.

### Conflict of interest statement

The author declares that the research was conducted in the absence of any commercial or financial relationships that could be construed as a potential conflict of interest.

## Note added in proof

The earlier demonstrations by the author of soma-to-germline inheritance should have been cited (Gorczynski and Steele, 1980, 1981). Further, a recent paper was overlooked in referencing. This demonstrated the soma-to-germline feedback phenomenon for regulatory double stranded RNA triggering RNA interference (RNAi) in *C. elegans* (Devanapally et al., 2015).
